# Alpha-helices as alignment reporters in residual dipolar coupling analysis of proteins

**DOI:** 10.1007/s10858-024-00456-5

**Published:** 2024-12-11

**Authors:** Yang Shen, Marshall J. Smith, John M. Louis, Ad Bax

**Affiliations:** Laboratory of Chemical Physics, National Institute of Diabetes and Digestive and Kidney Diseases, National Institutes of Health, Bethesda, MD 20892-0520 USA

**Keywords:** RDC, Protein dynamics, Protein NMR, Domain motion, SARS-CoV-2 MPro

## Abstract

Inclusion of residual dipolar couplings (RDCs) during the early rounds of protein structure determination requires use of a floating alignment tensor or knowledge of the alignment tensor strength and rhombicity. For proteins with interdomain motion, such analysis can falsely hide the presence of domain dynamics. We demonstrate for three proteins, maltotriose-ligated maltose binding protein (MBP), Ca^2+^-ligated calmodulin, and a monomeric N-terminal deletion mutant of the SARS-CoV-2 Main Protease, MPro, that good alignment tensor estimates of their domains can be obtained from RDCs measured for residues that are identified as α-helical based on their chemical shifts. The program, Helix-Fit, fits the RDCs to idealized α-helical coordinates, often yielding a comparable or better alignment tensor estimate than fitting to the actual high-resolution X-ray helix coordinates. The 13 helices of ligated MBP all show very similar alignment tensors, indicative of a high degree of order relative to one another. By contrast, while for monomeric MPro the alignment strengths of the five helices in the C-terminal helical domain (residues 200–306) are very similar, pointing to a well-ordered domain, the single α-helix Y54-I59 in the N-terminal catalytic domain (residues 10–185) aligns considerably weaker. This result indicates the presence of large amplitude motions of either Y54-I59 or of the entire N-terminal domain relative to the C-terminal domain, contrasting with the high degree of order seen in the native homodimeric structure.

**Supplementary Information:**

The online version contains supplementary material available at 10.1007/s10858-024-00456-5.

## Introduction

Residual dipolar couplings (RDCs) in biological macromolecules can be observed by solution NMR when there exists a net, time-averaged orientation of the molecule relative to an external magnetic field (Tolman et al. 1995; Tjandra and Bax 1997). These RDCs can yield very precise information on the time-averaged orientation of bonds relative to the global reference frame of the molecule’s alignment tensor, thereby encoding both structural and dynamic information (Bertini et al. 2004; Lindorff-Larsen et al. 2005; Lange et al. 2008).

Weak molecular alignment relative to the magnetic field can be obtained by multiple mechanisms, including paramagnetism (Tolman et al. 1995; Wang et al. 2020), dilute liquid crystalline phases (Tjandra and Bax 1997; Clore et al. 1998a, b; Hansen et al. 1998; Ruckert and Otting 2000), and anisotropically compressed hydrogels (Sass et al. 2000; Tycko et al. 2000). However, such measurements are often restricted to a single mode of alignment because optimizing each alignment method can be labor intensive. However, once a suitably aligned sample for RDC measurements has been generated, it is relatively straightforward to measure not only the most commonly used ^1^D_NH_ RDCs, but also many additional ^1^H-^13^C, ^13^C-^15^N, and ^13^C-^13^C dipolar couplings (Tjandra and Bax 1997; Yang et al. 1999; Evenas et al. 2001b; Chiliveri et al. 2022). The latter can agree better with crystallographically determined reference structures because they are not impacted by errors in the precise positions of the amide hydrogen atoms, which typically are added to the X-ray crystal structure by assuming standard, in-plane geometry (Ottiger and Bax 1998).

The strong dependence of RDCs on bond vector orientation can make it difficult to effectively use them during early rounds of structure calculations because it often causes very large scatter between experimental RDCs and values predicted for imperfect structural models. Either a floating alignment tensor may be used during the simulated annealing process (Sass et al. 2001), or a rough estimate of the alignment tensor’s magnitude and rhombicity can be obtained from a histogram analysis of the distribution of RDCs (Clore et al. 1998a, b). Even when an approximate protein structure is available it may be difficult to accurately define its alignment tensor or to evaluate whether the protein exists in solution as a single globular unit, or as a set of flexibly linked domains. Poor fits of the RDCs to molecular coordinates also can be caused by experimental errors, e.g. assignment errors or RDCs derived from partially overlapped resonances. Alternatively, a poor fit can result from dynamics in solution that is not reflected in the reference structure, or from true differences between solution and crystalline states. It therefore is desirable to identify self-consistent RDCs that correspond to a well-defined local alignment tensor, or multiple well-defined tensors for different regions in a protein that contains flexibly linked domains.

Here, we demonstrate that RDCs in α-helices, which are identified from backbone chemical shifts by the TALOS-N program (Shen and Bax 2013), can yield high quality alignment tensor information, provided that multiple types of backbone RDCs are available per residue. We introduce the program Helix-Fit, which relies on the assumption of idealized helical coordinates, and often yields a quality for the RDC fit that is comparable to using the high-resolution X-ray coordinates of such helices. Use of Helix-Fit is demonstrated for three proteins, maltose binding protein (MBP), calmodulin, and a monomeric form of the main Protease (MPro) of SARS-CoV-2.

The accuracy of alignment tensors depends on how well orientations are sampled by the internuclear vectors for which RDCs are measured. Fushman et al. introduced a “generalized sampling parameter”, Ξ, that quantifies the distribution of vector orientations on a scale from 0 (optimal sampling) to 1 (poor sampling) (Fushman et al. 2000). Whereas for N-H vectors in α-helices this sampling is poor (Ξ ≈ 0.83), excellent sampling (Ξ ≤ 0.17) applies for other vectors (C^α^-C′; N-C′; C^α^-H^α^; C′-H^N^; and C′-H^α^) whose RDCs are readily measured. The favorable orientational sampling by these vectors benefits the robustness at which alignment tensors of α-helices can be determined (Fushman et al. 2000).

## Results

### Identification of α-helices

When atomic coordinates are available, α-helices are characterized by backbone torsion angles, typically falling in a relatively narrow range (ϕ = − 63 ± 6°; ψ = − 42 ± 6°), and their H-bonding between the C=O of residue *i* and the H^N^ of *i* + 4 (Kabsch and Sander 1983). In the absence of structural information, α-helices are readily identified by their characteristic deviations from random coil chemical shifts (Spera and Bax 1991; Wishart et al. 1991) or, if available, sequential NOE patterns (Wüthrich 1986). The H-bonding pattern in α-helices results in highly regular structures that often closely fit to an idealized α-helix (Table S1 for ideal helical parameters used in this study). As a consequence, RDCs for backbone N-H pairs, whose internuclear vectors differ by 15.8° in orientation from the helix axis, show a characteristic sinusoidal pattern as a function of residue number with a periodicity of 3.6 residues, known as the “dipolar wave” (Mesleh et al. 2003). Below, we used the neural-network based TALOS-N program (Shen and Bax 2013) for identifying α-helices of ≥ 5 residues in length. For our current purpose of finding close-to-ideal α-helices, we found it necessary to terminate helices at the last residue preceding a Pro residue, or to start them at the first residue following a Pro when such a residue is embedded in a longer, kinked α-helix.

### RDC fitting of α-helices

Agreement between RDCs and atomic coordinates is limited by the precision at which RDCs can be measured as well as by uncertainties in the atomic coordinates. The latter often dominate the scatter between observed and best-fitted RDCs, obtained from a singular value decomposition (SVD) fit (Losonczi et al. 1999) of the RDCs to the internuclear vector orientations (Shen et al. 2023). The SVD fit can include weighting to account for the experimental error estimates, but in our examples below, the experimental errors are assumed to scale with the inverse of the dipolar interaction constants that are determined by the magnetogyric ratios and internuclear distances of the pairs of atoms. For example, the errors in ^1^D_C′Cα_ are assumed to be five times smaller than for ^1^D_NH_, effectively giving ^1^D_C′Cα_ and ^1^D_NH_ equal weight in the analysis. The use of equal weights for fitting the various types of normalized RDCs, available for MBP, calmodulin, and monomeric MPro, is justified by comparable qualities of their SVD fits, indicating that the errors in the fit are dominated by coordinate uncertainty, not by measurement error of the RDCs. However, in the program Helix-Fit, as well as all our other RDC analysis software, the estimated error in the measured RDC will be used if specified in the input table.

For a well-ordered globular domain that contains multiple helices, separate fits of the RDCs to its various helices are expected to yield very similar alignment tensors, with the uncertainty in each alignment tensor determined by jackknifing. The jackknife procedure cyclically omits one RDC from the total set of N RDCs, and then carries out N SVD fits on the remaining N-1 RDCs, resulting in N alignment tensors. Similarly, jackknifing at the residue level can be carried out by cyclically leaving out all RDCs (e.g. ^1^D_NH_, ^1^D_C′N_, ^1^D_CαC′_, ^2^D_HC′_) measured for a given amide, a procedure useful for identifying whether a residue identified by TALOS-N as the terminal helix residue indeed is consistent with helical geometry.

We represent the uncertainty in the orientation and rhombicity of the final, averaged alignment tensor, < *S* > , by ε(*S*), which is related to the spread in their normalized scalar products (Sass et al. 1999):

1a $$\varepsilon \left( S \right) = \left( {\text{N}} \right)^{{{1}/{2}}} {\text{RMS}}\left[ {\left\{ {{1} - {\text{P}}\left( {S_{i} ,\, < S > } \right)} \right\}} \right]$$

where RMS{1-P(*S*_*i*_*,* < *S* >)} =$\sqrt{\sum_{i=1..N}{\left(1-\text{P}({S}_{i},\langle S\rangle )\right)}^{2}/N}$ is the root-mean-square (RMS) deviation from unity for the normalized scalar products P(*S*_i_, < *S* >) of the N jackknifed five-dimensional Saupe matrix vectors, *S*_*i*_ = [*S*_zz_, (1/$\sqrt{3}$)(*S*_xx_-*S*_yy_), (2/$\sqrt{3}$)(*S*_xy_), (2/$\sqrt{3}$)(*S*_xz_), (2/$\sqrt{3}$)(*S*_yz_)]_i=1..N_, relative to the averaged Saupe matrix vector < *S* > , or P(*S*_i_, < *S* >) = *S*_i_· < *S* > /(|*S*_i_| ×|< *S* >|).

Analogously, the fractional uncertainty in the alignment strength is

1b $$\varepsilon \left( G \right) = \left( {\text{N}} \right)^{{{1}/{2}}} {\text{RMS}}\left( {G_{i} - \, < G > } \right)/ < G >$$

with *G*_*i*_ given by (Clore and Garrett 1999):

1c $$G_{i} = \left\{ {D_{a,i}^{{2}} \left[ {{4} + {3}Rh_{i}^{{2}} } \right]/{5}} \right\}^{{{1}/{2}}}$$

where *D*_*a,i*_ and *Rh*_*i*_ are the magnitude and rhombicity of the alignment tensor, obtained when RDC *i* is removed from the fit. The fit is repeated *N* times and each SVD fit omits a different coupling from the *N* available RDCs. < *G* > represents the average over the *N G*_*i*_ values obtained by this jackknife procedure, with reported values normalized to the interaction strength of the ^15^N-^1^H backbone amide pair.

A jackknifed *Q*_*jk*_ factor (Shen et al. 2023) is used to evaluate the SVD fit:

2 $${Q}_{jk}=\sqrt{\sum_{i=1..N}\frac{({{{W}_{pq}D}_{i}^{pred}-{W}_{pq}{D}_{i}^{meas})}^{2}}{N\left\{{D}_{a,i}^{2}\left(4+3{Rh}_{i}^{2}\right)/5\right\}}}$$

where ${D}_{i}^{pred}$ and ${D}_{i}^{meas}$ refer to the predicted and the measured values for coupling *i* when the SVD is carried out for all other *N* − 1 couplings, excepting *i*. When fitting a large number (N >> 5) of RDCs, *Q*_*jk*_ approaches the standard *Q* value, derived when including all RDCs in the SVD fit. However, because the SVD fit includes five adjustable parameters, the standard method for deriving *Q* strongly overestimates the goodness of the fit when the number of RDCs is small (N <  ~ 25). This problem is solved by the computationally more burdensome jackknifing procedure.

With five adjustable parameters in the SVD fit, the jackknifing procedure requires a minimum of six RDCs. For more robust results, in practice we only consider helices with at least eight RDCs. Therefore, if only ^1^*D*_NH_ RDCs are measured, helices are required to be relatively long for such an analysis. However, since several other backbone RDCs can be obtained at high relative precision, we limit the minimum length of α-helical elements evaluated by Helix-Fit to five residues, *i.e.* including at least one *i to i* − 4 amide-to-carbonyl H-bond.

Helix-Fit can use either the atomic coordinates of the reference structure for SVD fitting or, controlled by a flag, uses the coordinates of an idealized helix that is best-fit superimposed on the heavy backbone atoms (N, C^α^, and C′) of the residues selected.

### Maltose binding protein

**Fig. 1:**
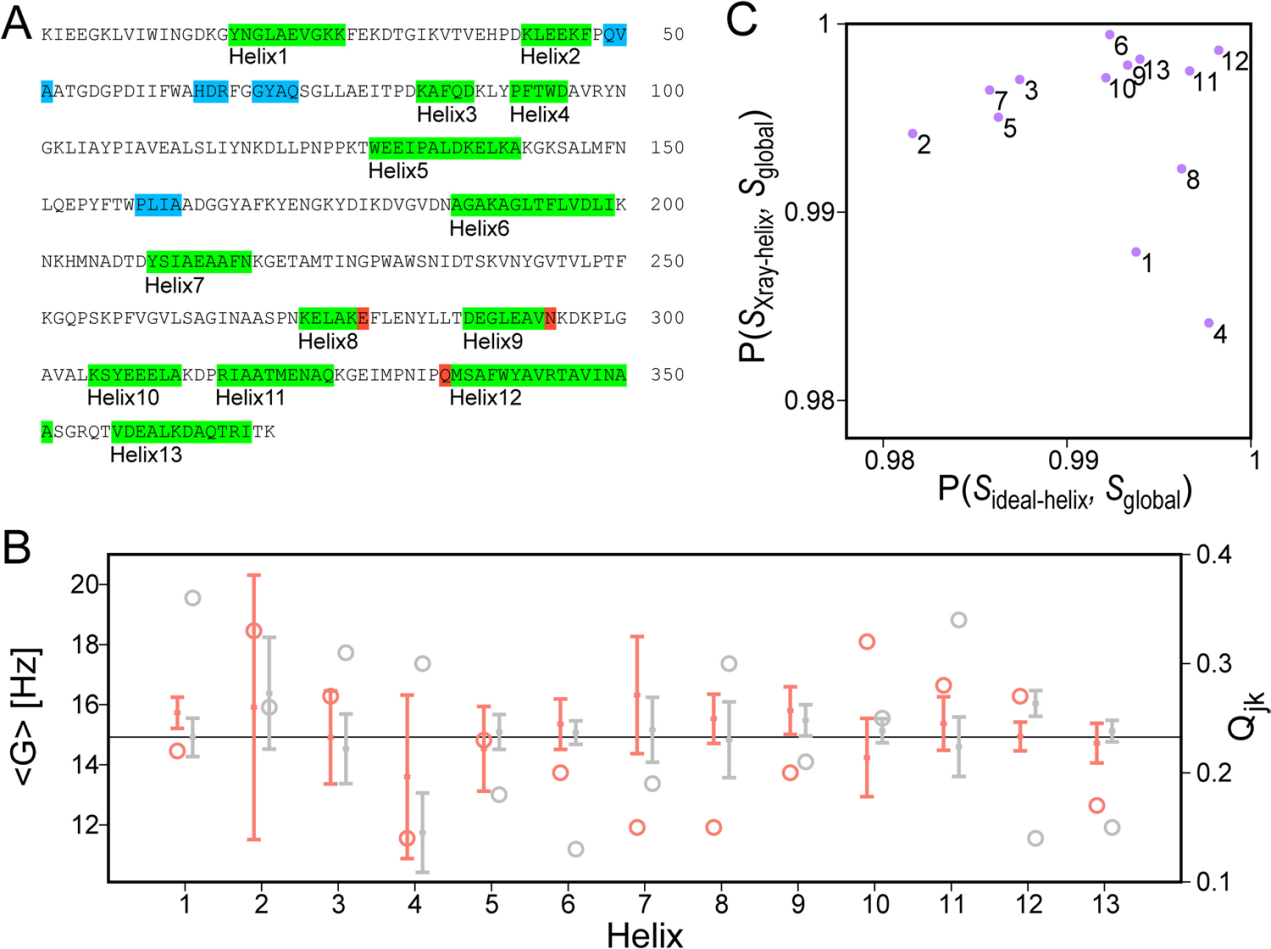
^1^D_HN_, ^1^D_C′N_ and ^1^D_CαC′_ RDC analysis of α-helices in MBP. The generalized sampling parameter for these couplings is Ξ = 0.066. **A** Colored bars along the amino acid sequence refer to residues defined as helical by TALOS-N. Only helices with length ≥ 5 residues are used for RDC analysis, with some terminal residues (red) culled prior to final fitting due to their RDC incompatibility with α-helical structure. Helices < 5 residues are marked in blue and were not considered in the Helix-Fit analysis. **B** *G* values and their jackknifed standard deviations for the 13 helices in maltotriose-ligated MBP obtained when using idealized α-helical coordinates (orange) and X-ray atomic coordinates (PDB entry 3MBP, gray); the *G* value obtained when fitting all RDCs is plotted as the horizontal line, and the corresponding *Q*_*jk*_ values (open circles) correspond to the scale at the right side of the panel. **C** Correlation of normalized scalar products between the alignment tensors obtained for the individual α-helices (labeled by helix number) and the tensor obtained from the full set of RDCs (*S*_global_), covering the entire protein

For ligated maltose binding protein (MBP), there is close agreement between α-helices identified from crystallographic atomic coordinates (PDB entry 3MBP) (Quiocho et al. 1997) and from NMR backbone chemical shifts (BMRB entry 4354) (Gardner et al. 1998) (Fig. 1A). Small differences are confined to the N- and C-termini which may be recognized as helical by TALOS-N, even though their respective ϕ and ψ angles sometimes deviate by more than 30° from canonical helix values (Fig. 1A). For RDC-fitting purposes, we therefore evaluate whether removal of RDCs that report on the orientation of the N-terminal and C-terminal amide planes, identified as helical by TALOS-N, can greatly improve the quality of the RDC fit to the coordinates of an idealized helix (see Methods). For example, the Q_jk_ value obtained from fitting helix 12 decreases from 0.53 to 0.33 after removal of the RDCs arising from the N-terminal amide plane of Q335; a similar improvement of Q_jk_ from 0.30 to 0.19 is obtained for helix 8 after removing the RDCs related to the C-terminal amide plane of E281. The *G* values and their standard deviations can be compared for fits of the RDCs to the ideal helical coordinates and those in the actual X-ray structures (Fig. 1B). As can be seen, for MBP the spread in *G* values is small, and the normalized scalar products between the global alignment tensor of the entire protein, *S*_Global_, and fits to tensors obtained for the individual X-ray (*S*_Xray-helix_) or idealized (*S*_ideal-helix_) α-helices are very large (Fig. 1C), confirming that the helices in ligated MBP are highly ordered.

### Calmodulin

Calmodulin is a two-domain protein, regulating the activity of more than one hundred targets, many of them kinases, in a Ca^2+^-dependent manner (Crivici and Ikura 1995). It contains two globular domains that are homologous in sequence, and each consists of two EF-hand Ca^2+^-binding motifs. The two domains are connected by a linker that is entirely α-helical in the crystalline state (Babu et al. 1988; Wilson and Brunger 2000). However, based on solution ^15^N relaxation analysis that showed independent, near-isotropic rotational diffusion of its two domains, this so-called “central helix” is highly disordered near its midpoint (Barbato et al. 1992). As expected, binding of a paramagnetic lanthanide in one of the two N-terminal Ca^2+^-binding sites imposes substantial magnetic field alignment for the N-terminal domain, but much weaker alignment on the flexibly linked C-terminal domain (Bertini et al. 2004).

**Fig. 2:**
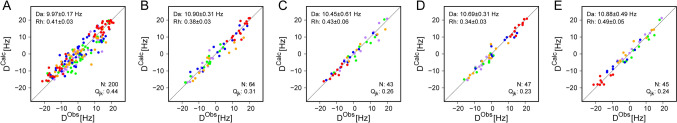
RDC analysis of α-helices in Ca^2+^-calmodulin’s N-terminal domain. **A** Fit of the normalized ^1^D_NH_ (red ball), ^1^D_HαCα_ (yellow ball), ^1^D_C′N_ (green ball), ^1^D_CαC′_ (blue ball), and ^2^D_HαC′_ (pink ball) RDCs, reported by Chou et al. (2001) against values predicted by an SVD fit to the 1 Å X-ray structure (PDB entry 1EXR) (Wilson and Brunger 2000). **B**–**E** Individual SVD fits of normalized experimental RDCs to coordinates of idealized α-helices, best-fit superimposed on the corresponding backbone atoms (N, C^α^, C′) of the X-ray coordinates. **B** Helix 1 (E6-F19); **C** Helix 2 (T29 to S38); **D** Helix 3 (E45–E54); and **E** Helix 4 (F65-R74). *N* refers to the number of backbone RDCs available for the SVD fits, and *Q*_*jk*_ to the jackknifed *Q* values

A large set of ^1^H-^15^N, ^1^H^α^-^13^C^α^, ^13^C′_i−1_-N_i_ and ^13^C^α^-^13^C′, as well as two-bond ^1^H^α^-^13^C′ RDCs were previously reported for Ca^2+^-ligated calmodulin (Chou et al. 2001). For each calmodulin domain, the RDC data pointed to an, on average, narrower target-binding groove in solution than seen in the X-ray structure. Indeed, a best-fit of the RDCs in calmodulin’s four N-terminal domain α-helices to the 1 Å X-ray structure shows a rather poor fit (Q_*jk*_ = 0.44) (Fig. 2A). Fitting the same RDCs to the experimental X-ray structure but with coordinates of each of the four helices replaced by best-fit superimposed ideal helices shows a comparably poor fit (Q_*jk*_ = 0.43). However, separately fitting the RDCs to coordinates of individual idealized helices that are best-fit superimposed on the X-ray helices are considerably better (Fig. 2B–E), comparable to results obtained by fitting to the 1.0 Å X-ray coordinates of these four helices (SI Fig. S1). This result is consistent with the earlier observation that the average EF-hand interhelical angles in solution differ from those seen in the X-ray structure by *ca* 25° (Chou et al. 2001).

**Fig. 3:**
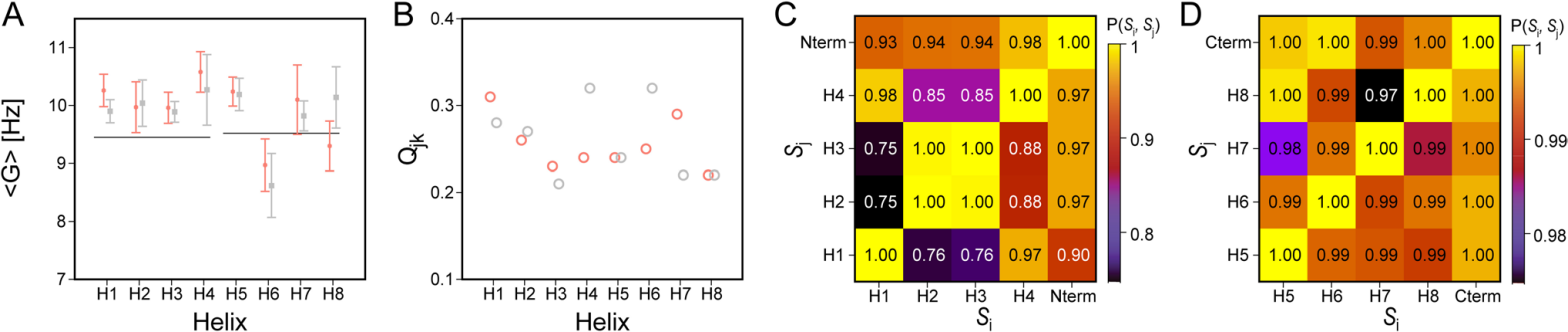
Alignment of helices in Ca^2+^-ligated calmodulin, oriented in 15 mg/ml Pf1 (Chou et al. 2001). **A** Generalized alignment strengths, *G*, obtained by eight separate SVD fits of its α-helices (E6-F19; T29-S38; E45-E54; F65-R74; E82-F92; A102-N111; D118-E127; Y138-M145) when using the X-ray structure (1EXR; grey) or coordinates of idealized helices that are best-fitted to the X-ray structure (orange). ^1^D_NH_, ^1^D_HαCα_, ^1^D_C′N_, ^1^D_CαC′_, and.^2^D_HαC′_ couplings were used (Ξ = 0.00). Error bars correspond to the ε(*G*) values derived by jackknifing (Eq. 1b). The left horizontal line corresponds to the <*G*> value obtained by simultaneously fitting RDCs of all four N-terminal domain helices to the X-ray structure (*D*_*a*_ = 9.97 ± 0.17 Hz; *Rh* = 0.41 ± 0.03;* G* = 9.46 ± 0.15); the right horizontal line corresponds to the <*G*> value obtained by fitting RDCs of all four C-terminal domain helices to the X-ray structure (*D*_*a*_ = 9.27 ± 0.17 Hz; *Rh* = 0.65 ± 0.02;* G* = 9.52 ± 0.19). **B** *Q*_*jk*_ values obtained by fitting RDCs to the X-ray (grey) and idealized (orange) helical coordinates. **C** Normalized scalar product values P(*S*_i_, *S*_j_) for the alignment tensors *S* obtained for N-terminal domain α-helices relative to one another, and relative to the alignment tensor obtained for a global RDC fit of the entire domain (i,j = ‘H1’, ‘H2’, ‘H3’, ‘H4’, and ‘Nterm’). The lower right half corresponds to use of idealized helical coordinates; the top left half corresponds to using X-ray coordinates. **D** Same as **C** but for the C-terminal domain (see also SI Fig. S2)

RDC fits of the four individual N-terminal domain helices all exhibit *G* values well above the *G* value obtained when fitting RDCs of the entire N-terminal domain, regardless of using idealized coordinates or the X-ray coordinates for the SVD fits (Fig. 3A). This discrepancy in *G* values is primarily caused by a difference between the average relative helix orientations seen in the X-ray structure and those present in solution (Chou et al. 2001). These differences in average helix orientation are reflected in the well below unity value of the normalized scalar products of the alignment tensors obtained for the individual helices, both relative to one another and relative to a fit of each entire domain (Fig. 3B, C). Indeed, the variability in the interhelical EF-hand angles seen in high-resolution X-ray structures of complexes with different target sites highlights the importance of the flexible nature of these EF-hands, which allows calmodulin to fine-tune the interactions with its wide range of targets (Akke and Chazin 2001).

As previously reported (Chou et al. 2001), RDCs measured for calmodulin’s C-terminal domain are more consistent with the X-ray structure than those of the N-terminal domain, simply reflecting a smaller difference in the average EF-hand interhelical angles in solution relative to the crystalline state. This conclusion is also reflected in P(*S*_i_, *S*_j_) values for the C-terminal domain helices (i,j = ‘H5’ to ‘H8’) (Fig. 3D) that are closer to unity than for the N-terminal domain (i,j = ‘H1’ to ‘H4’) (Fig. 3C). Remarkably, the *G* values of the N- and C-terminal domains are very similar to one another, despite the fact that these domains are flexibly linked. While the P(*S*_Nterm_, *S*_Cterm_) value for the alignment tensors of the N- and C-terminal domains, *S*_Nterm_ and *S*_Cterm_, is low (0.08) when using the X-ray structure as a reference, reorienting the C-terminal domain such that its principal axis system coincides with that of the N-terminal domain raises this P(*S*_Nterm_, *S*_Cterm_) value to 0.98 (Fig. S3). It could be argued that the latter result is consistent with a static structure that adopts this alternate relative domain orientation. This incorrect conclusion highlights the fact that a P(*S*_Nterm_, *S*_Cterm_) value near unity does not prove the absence of large angular motions; however, a low P(*S*_Nterm_, *S*_Cterm_) value reflects a difference in static structure and/or relative domain motions. A statistically meaningful difference in *G* value for different domains requires interdomain motion, typically of substantial angular amplitude. For randomly oriented pairs of alignment tensors with random rhombicity, [*S*_i_, *S*_j_], the P(*S*_i_, *S*_j_) distribution spans the range of 0 to 1, with the relative probability of a P(*S*_i_, *S*_j_) = α value approximately decreasing with cos(2α/π) (SI Fig. S4).

### Monomeric SARS-CoV-2 MPro

**Fig. 4:**
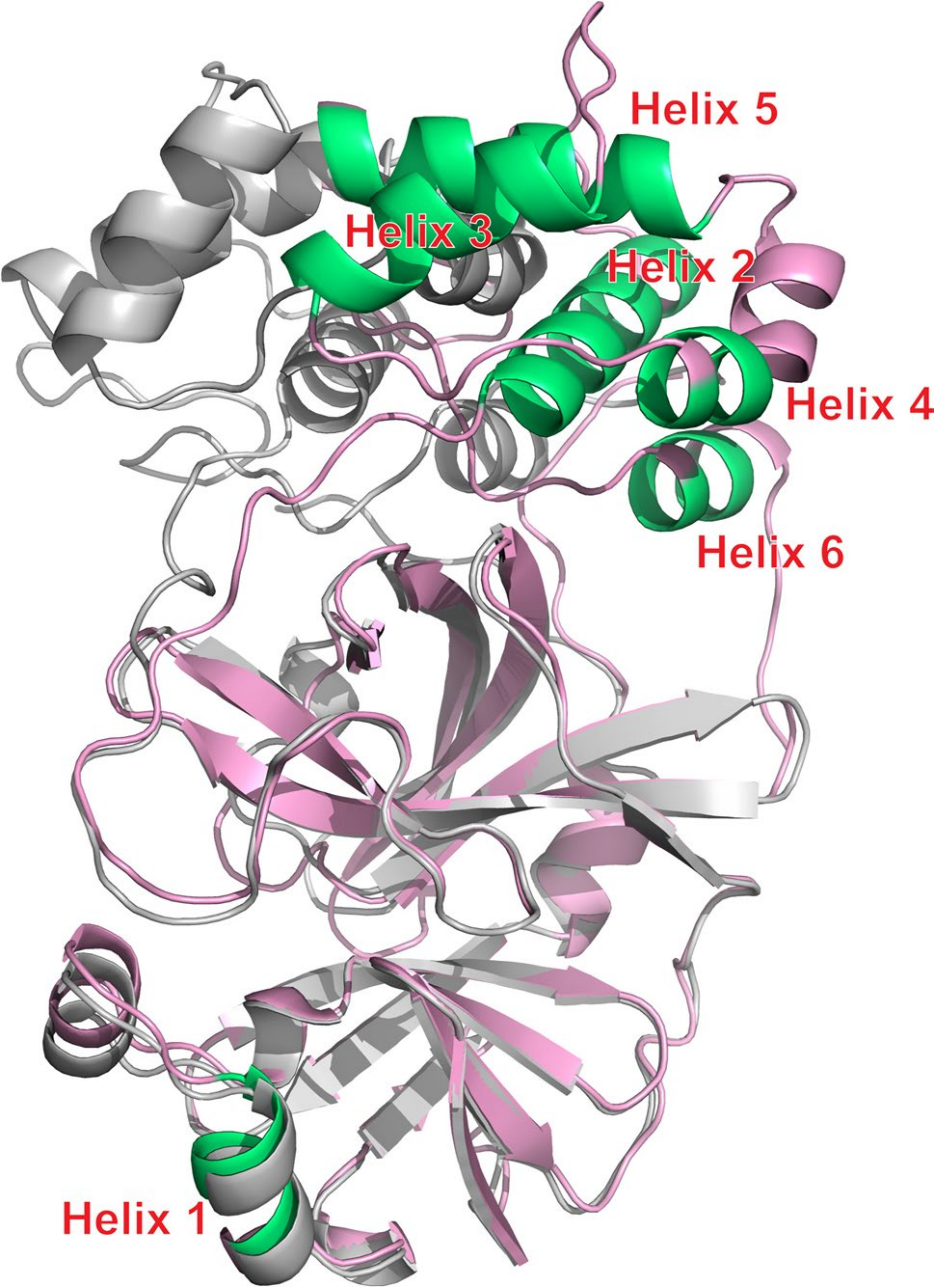
Superposition of MPro subunit taken from the native homodimer X-ray structure, 7K3T (pink), and its R298A monomeric mutant, 2QCY (grey). The six 7K3T helices used for RDC analysis are shown in green

**Fig. 5:**
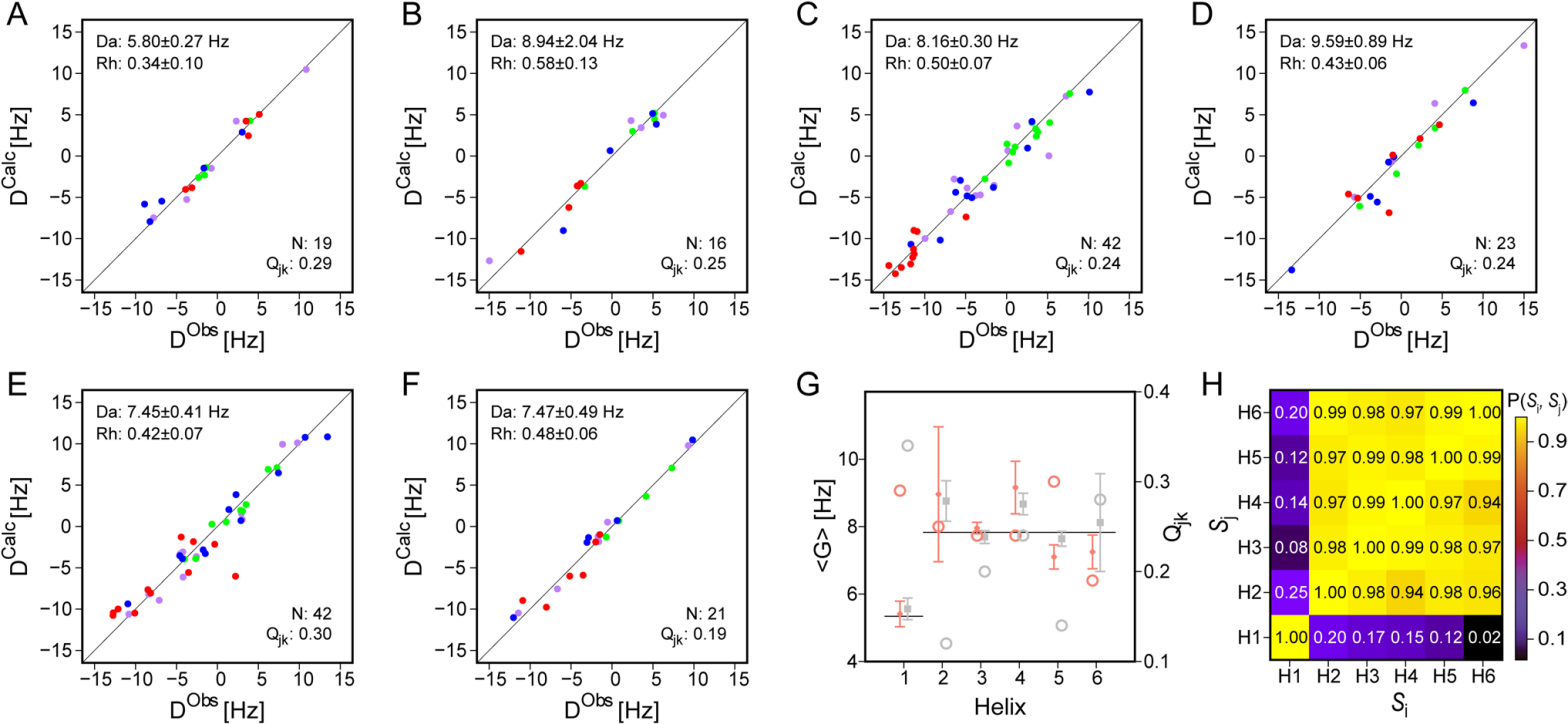
RDC analysis of α-helices in monomeric SARS-CoV-2 MPro^10−306,H41Q^. Fits of the normalized ^1^D_NH_ (red ball), ^1^D_C′N_ (green ball), ^1^D_CαC′_ (blue ball), and.^2^D_HNC′_ (pink ball) RDCs to the X-ray coordinates of homodimeric MPro (PDB entry 7K3T) are shown for the following helices: **A** Y54-I59; **B** T201-I213; **C**L227-Y237; **D** D245-L250; **E** V261-Q273; and **F** F294-Q299. The generalized sampling parameter for these couplings, Ξ, is 0.062. **G** *G* values obtained for the six helices when using 7K3T (grey) or idealized helices (orange) as reference structures; the reference <*G*> value of 7.83 obtained for the five C-terminal helices (Helix-2 to Helix-6), and < *G* >  = 5.34 obtained for Helix-1 in the N-terminal domain, are plotted as horizontal lines. *Q*_*jk*_ values (open circles) correspond to the scale at the right side of the panel. **H** Normalized scalar products P(*S*_*i*_*, S*_*j*_) for all pairs of the alignment tensors of six helices, [*S*_i_, *S*_j_] (i,j = “H1” to “H6”), obtained when fitting the helical RDCs to the coordinates of the homodimeric X-ray structure (7K3T) (upper-left half), and ideal helices (lower-right half). For the corresponding P(*S*_i_, *S*_j_) values when using the monomeric MPro X-ray structures (2QCY; 2PWX; 3F9E), see SI Fig. S6

A SARS-CoV-2 MPro construct, lacking the N-terminal residues S1-P9 that stabilize the dimerization of the native form of the enzyme, and further inactivated by the active site H41Q mutation (MPro^10−306,H41Q^), was expressed in D_2_O medium. For multiple β-sheet residues in the N-terminal domain and α-helical residues in the C-terminal domain, back exchange of the amide protons with the protonated solvent was incomplete, even after several days at neutral pH and room temperature, causing multiple backbone amide signals to be very weak or absent. Nevertheless, a sufficiently large set of ^1^D_NH_, ^1^D_C′N_, ^1^D_CαC′_, and ^2^D_C′H_ RDCs was obtained (Table S2) that yielded well-defined alignment tensors for each of its six α-helices: one in the N-terminal domain (Y54-I59) and five in the C-terminal domain (R201-I213; L227-Y237; D245-L250; V261-Q273; and F294-Q299 (Fig. 4). There are several X-ray structures for monomeric MPro (2QCY (Shi et al. 2008); 2PWX (Chen et al. 2008); and 3F9E (Hu et al. 2009). These all pertain to the highly homologous (~ 96% identity, Zhang et al. 2011) previous SARS coronavirus isolate and display a different orientation of the C-terminal domain (I200-Q306) relative to the N-terminal catalytic domain from that seen in the native dimeric state (Fig. 4). As was observed for calmodulin, good SVD fits were obtained for RDCS of the individual helices measured in the monomeric state to coordinates of helices in the 1.2-Å X-ray structure of the native dimer, 7K3T (Andi et al. 2022) (Fig. 5A–F). On average, slightly lower quality fits and less well-defined alignment tensors, reflected in higher *Q*_*jk*_ and ε(*G*) values, were obtained for fits to idealized helices (Fig. 5G).

**Fig. 6:**
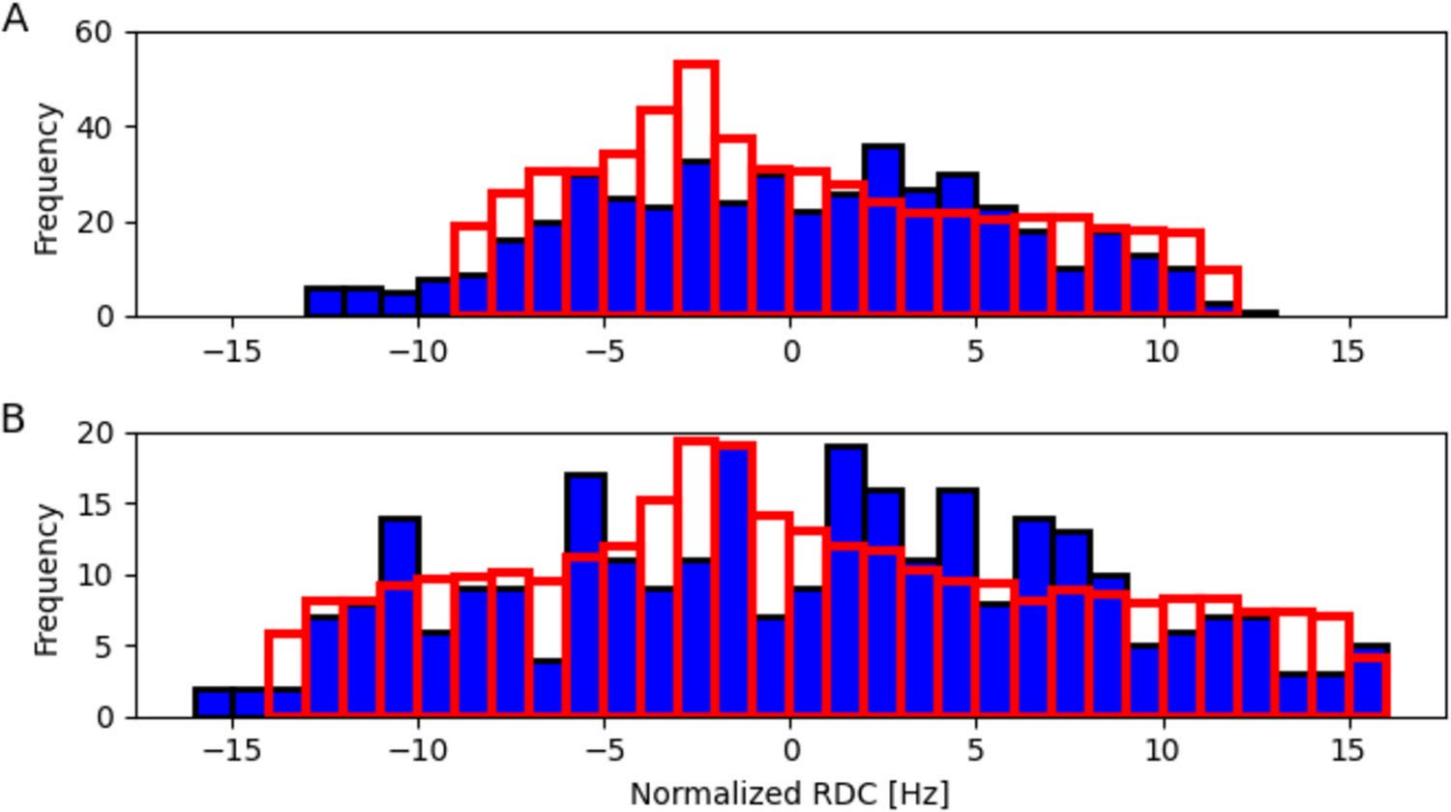
Histogram distributions of backbone RDCs in monomeric MPro, normalized to ^1^D_NH_. **A** Experimental RDC distribution for the N-terminal domain in blue, and in red the histogram expected for uniformly distributed vector orientations and the alignment tensor obtained for helix Y54-I59. **B** Analogous plot of C-terminal domain RDCs, and the expected histogram distribution if vectors were uniformly distributed with an alignment strength and rhombicity obtained from simultaneously fitting all helical RDCs in this domain

Remarkably, the generalized alignment magnitude, *G*, is considerably larger for the C-terminal domain than for the Y54-I59 helix in the N-terminal domain, indicative of large amplitude motions of this helix or of the entire N-terminal domain relative to the C-terminal domain (Fig. 5G). Although assignments for the N-terminal domain remain incomplete due to the above mentioned slow back exchange as well as conformational exchange broadening, a large number of yet to be fully identified RDCs was measured for the N-terminal domain. The distributions of normalized RDCs seen in the N-terminal domain appears considerably narrower than for the C-terminal domain (Fig. 6), pointing to a lower alignment strength (Clore et al. 1998a, b). However, the histogram RDC distribution expected for the N-terminal domain when using the alignment tensor obtained for its Y54-I59 helix is somewhat narrower than the observed distribution (Fig. 6A), suggesting that the helix orientation undergoes dynamic fluctuations relative to the N-terminal domain.

For the C-terminal domain, its helices show very similar alignment strengths, indicative of a well-ordered domain which is consistent with the slow hydrogen exchange rates seen for many of its backbone amides. Mobility of helix Y54-I59 relative to the C-terminal domain differs strongly from what is observed when evaluating the RDCs previously measured for the homodimeric state (Robertson et al. 2021), which show very similar *G* values and high P(*S*_i_, *S*_j_) values (i,j = ‘H1’ to ‘H6’) (Fig. S5).

For RDCs measured here in the monomeric state and for all of the X-ray structures evaluated (2QCY; 2PWX; 3F9E; and dimeric 7K3T), we find a low value of the normalized scalar products, P(*S*_i_, *S*_j_), of the alignment tensor of the Y54-I59 helix (*S*_i_, i = ’H1’) relative to those of the C-terminal domain helices (*S*_j_, j = ‘H2’ to ‘H6’) (Fig. 5H; SI Fig. S6), confirming that in solution the relative domain orientation differs from that in any of the X-ray structures and presumably is subject to large amplitude dynamics.

For the C-terminal domain helices, the P(*S*_i_, *S*_j_) values (i,j = ‘H2’ to ‘H6’) are close to unity when using the 1.2 Å X-ray structure of the homodimer as a reference but, on average, somewhat lower when using the monomer X-ray structures as references (Fig. S6). However, superposition of the C-terminal domain of monomeric MPro X-ray structures on those of the homodimer shows close correspondence, with a C^α^ RMSD ≤ 0.64 Å. Therefore, these somewhat lower P(*S*_i_, *S*_j_) values likely result from slightly less accurate atomic coordinate positions in these monomeric X-ray structures.

## Discussion

Our RDC analysis of α-helical regions in proteins takes advantage of the highly regular backbone geometry imposed by the backbone H-bonding pattern in such helices. Consequently, RDCs fit about equally well to idealized α-helices and to the corresponding high-resolution X-ray coordinates, as reflected in comparable *Q*_*jk*_ values (Figs. 1B, 3B, 5G). If perturbations in the α-helical H-bonding pattern are present, they often are apparent in strong deviations from the expected secondary chemical shifts, and RDC fitting to idealized α-helical coordinates must be restricted to helices that display canonical ^13^C^α^, ^13^C^β^, and ^13^C′ secondary chemical shift values, and only to regions that do not include a Pro residue, which invariably kinks the helix axis.

RDC analysis of α-helices can provide insights into the dynamics of a system, even before either a full-fledged structure determination or a ^15^N NMR relaxation analysis has been completed. In practice, such an analysis requires that multiple RDCs per peptide group are measured because the uncertainty in alignment tensor parameters obtained for any given helix scales roughly with 1/√(N-5), where N is the number of measured RDCs per helix and 5 corresponds to the degrees of freedom in the SVD fit. Moreover, the orientational sampling of just N-H vectors in α-helices (Ξ = 0.83) typically will be insufficient for deriving accurate alignment tensors (Fushman et al. 2000). Even for the MPro dimer, two RDCs per amide (^1^D_NH_ and ^2^D_C′H_) were measured at high precision, despite the large size and slow tumbling of this 68 kD system (Robertson et al. 2021). For the monomeric MPro, which exhibits more favorable relaxation properties, four RDCs were measured per peptide plane (^1^D_NH_, ^1^D_C′N_, ^1^D_CαC′_, and ^2^D_HC′_). Even though, due to the planar geometry, these four couplings are partially redundant (Bryce and Bax 2004), their measurement errors are uncorrelated and the availability of all four couplings improves the reliability of the fits.

Our RDC analysis of maltotriose-ligated MBP showed that within experimental uncertainty, all its helices are subject to the same alignment tensor, confirming that in the ligated state this protein behaves as one globular, well-ordered system. RDC analysis of Ca^2+^-calmodulin revealed that the normalized scalar products between each of its four individual helices and the alignment tensor obtained for the entire N terminal domain falls well below unity, indicative of the previously identified substantial structural differences between the relative helix orientations of this domain in solution, (Chou et al. 2001) and in the X-ray structure. The calmodulin example also shows that the presence of essentially indistinguishable alignment tensor magnitudes, *G*, of its N- and C-terminal domains should not be misinterpreted as evidence for the absence of interdomain motion. Presumably as a result of the two domains’ very similar shape and charge distribution, their alignments in the Pf1 liquid crystal simply happen to be equally strong even while their relative orientation is highly dynamic (Barbato et al. 1992; Bertini et al. 2004).

Great interest exists in the monomeric structure of MPro because this protein is initially expressed as a polyprotein, and it needs to excise itself from this precursor before it can adopt the catalytically active homodimeric state (Tan et al. 2005; Shi et al. 2008; Nashed et al. 2022). Therapeutic strategies that target its ability to dimerize therefore may be powerful complements to traditional active site inhibitors (Goyal and Goyal 2020; Cantrelle et al. 2021; Berg et al. 2022). Even before assignments and NMR relaxation analysis of the monomeric MPro are complete, RDC analysis points to considerably weaker alignment of the active-site containing N-terminal domain than for the mostly α-helical C-terminal domain. Relative helix orientations in the C-terminal domain are largely consistent with those seen in both monomeric and homodimeric X-ray structures, but the relative orientation of the N- and C-terminal domains clearly does not match any of the X-ray structures, again pointing to interdomain dynamics. The presence of this interdomain motion is perhaps not surprising considering that the globular N-terminal domain retains much of its structure, with largely unchanged chemical shifts when expressed in isolation compared to values seen in the homodimeric state (Zhang et al. 2011; Robertson et al. 2022). Moreover, the linker between the N-and C-terminal domains in the dimeric protein is subject to considerable dynamic disorder, both in solution and in the crystalline state (Ebrahim et al. 2022; Shen et al. 2023). On the other hand, a 50-ns molecular dynamics trajectory revealed no significant changes in relative domain orientation for the monomeric state (Parmar et al. 2023), and a full RDC and ^15^N NMR relaxation analysis of monomeric MPro therefore has been initiated in our laboratory.

## Methods

RDC data for MBP are from (Evenäs et al. 2001a) and kindly provided to us by Dr. V. Tugarinov. Ca^2+^-calmodulin RDC data, recorded in 15 mg/mL Pf1 medium, were taken from (Chou et al. 2001).

Resonance assignments of the monomeric mutant MPro^10−306,H41Q^ (Nashed et al. 2022; Kovalevsky et al*.* 2024) were carried out using standard TROSY-based 3D triple resonance methods (Salzmann et al. 1999) on a perdeuterated, partially back-exchanged construct. Data were collected at 800 MHz for a 0.5 mM sample in 25 mM HEPES buffer at pH 6.9 with 20 mM NaCl, 1 mM TCEP, 3% D_2_O and 0.02% NaN_3_ at 15 °C. RDC data were collected at 25 °C on a 0.43 mM sample, with 11 mg/mL Pf1 used as the alignment medium. The resonance assignments were transferred from spectra acquired at 15 °C to those at 25 °C using multiple TROSY spectra collected over this temperature range.

^1^D_NH_ couplings were measured at 800 MHz, using the ARTSY method (Fitzkee and Bax 2010), with RDCs calculated from the cross-peak intensity ratios of two interleaved 2D ^1^H-^15^N TROSY spectra (Fitzkee and Bax 2010) for a total measurement time of 5 h for isotropic data and the same for the aligned sample. Additionally, two interleaved 3D ^1^H-^15^N TROSY-HNCO ARTSY spectra (17 h each) were collected at 600 MHz (Chiliveri et al. 2022). ^1^D_NC′_ and ^1^D_CαC′_ RDCs were measured at 800 and 600 MHz ^1^H frequencies, respectively, using 2D and 3D quantitative-J experiments (Chou et al. 2000; Jaroniec et al. 2004). Data recording time was 11 h for ^1^D_NC′_; 19 h for ^1^D_CαC′_, and similar for the measurement of ^1^J_NC′_ and ^1^J_CαC′_ under isotropic conditions. For improved precision of the extracted ^1^D_NC′_ and ^1^D_CαC′_ RDCs, data for the attenuated spectra were collected with six times more scans than the reference spectrum. ^2^D_C′H_ RDCs were measured at 600 MHz, using the 3D TROSY-AntiTROSY-Encoded RDC (TATER) method (Robertson et al. 2021). The total recording time was 18 h for each of the isotropic and aligned samples. Data processing was carried out using NMRPipe (Delaglio et al. 1995); assignments were carried out using CCPN-v3.1 software (Skinner et al. 2016), and RDC values were extracted using MATLAB scripts. RDC data used for analysis of the helices are included in SI Table S2 and RDC data for the full protein will be deposited in the BMRB once analysis has been completed.

Further details of the MPro^10−306,H41Q^ assignments and RDC data collection, carried out as part of a search to find the optimal RDC measurement methods for proteins of this intermediate size class, will be presented elsewhere.

### Software availability

The Helix-Fit program is available in server format at https://spin.niddk.nih.gov/bax/nmrserver/dc/helix_fit.html (requiring a generic google login) and will also be made available at NMRBox: https://nmrbox.nmrhub.org/.

## Supplementary Information

Below is the link to the electronic supplementary material.

Supplementary file1 (PDF 1334 KB)

## Data Availability

All data is included in the Supporting Information.
