# Supplementary material for: Alpha-helices as alignment reporters in residual dipolar coupling analysis of proteins
Source: J Biomol NMR. 2024 Dec 11;79(1):47–57. doi: 10.1007/s10858-024-00456-5 (PMC11832631; doi:10.1007/s10858-024-00456-5)
Supplement: Supplementary file 1 — Supplementary file1 (PDF 1334 KB) [file 10858_2024_456_MOESM1_ESM.pdf]

α-Helices as Alignment Reporters in Residual Dipolar Coupling Analysis of Proteins

Yang Shen, Marshall J. Smith, John M. Louis and Ad Bax

Laboratory of Chemical Physics, National Institute of Diabetes and Digestive and Kidney Diseases, National Institutes of Health, Bethesda, MD 20892-0520, U.S.A.

SUPPORTING INFORMATION

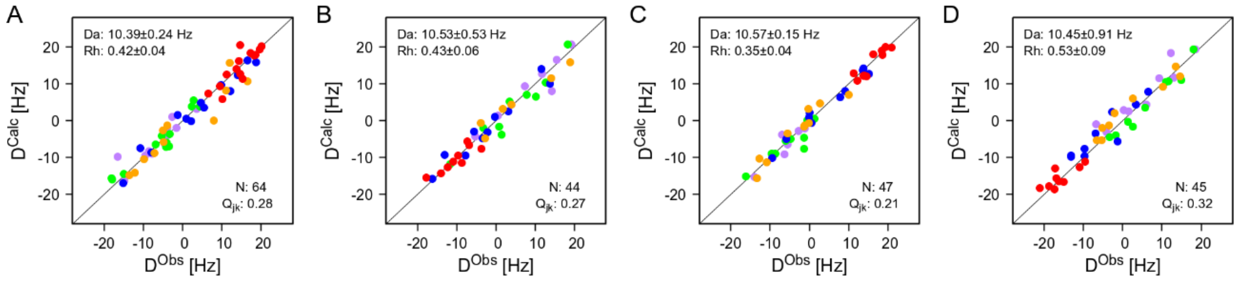

Figure S1. RDC analysis of α-helices in Ca^{2+}-calmodulin's N-terminal domain. Fit of the normalized $^1D_{NH}$ (●), $^1D_{H\alpha C\alpha}$ (●), $^1D_{C'N}$ (●), $^1D_{C\alpha C'}$ (●), and $^2D_{H\alpha C'}$ (●) RDCs, reported by Chou et al.(Chou *et al.* 2001) against values predicted by SVD fits to helices taken from the 1-Å X-ray structure (PDB entry 1EXR) (Wilson and Brunger 2000). (A) helix 1 (E6-F19); (B) helix 2 (T29 to S38); (C) helix 3 (E45 to E54); and (D) helix 4 (F65-R74).

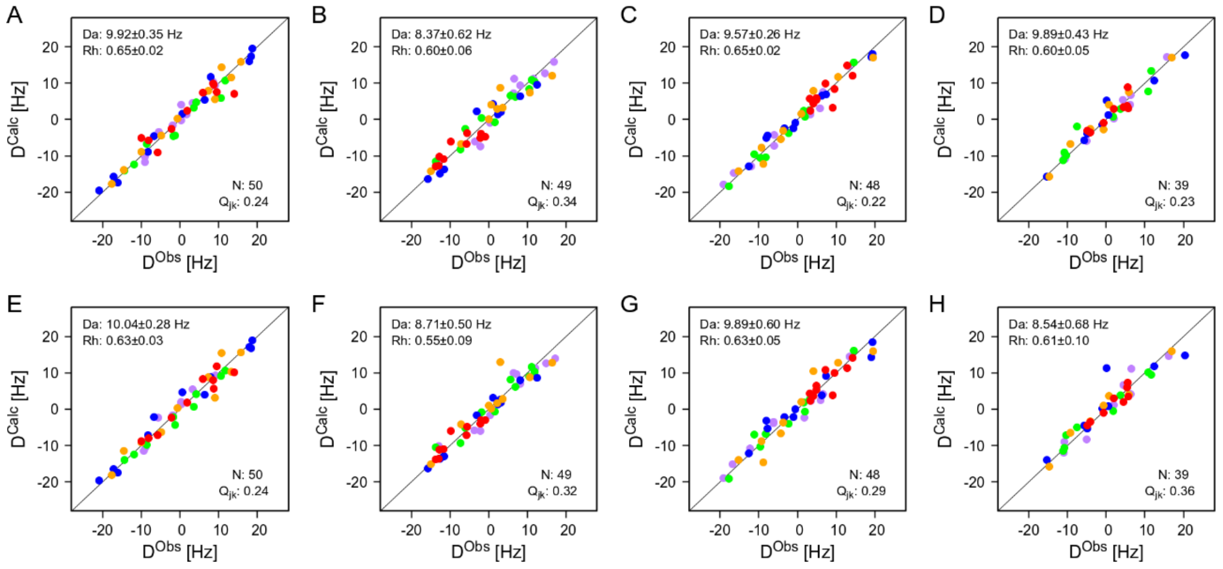

Figure S2. RDC analysis of α-helices in Ca^{2+}-calmodulin's C-terminal domain. Fit of the normalized $^1D_{NH}$ (●), $^1D_{H\alpha C\alpha}$ (●), $^1D_{C'N}$ (●), $^1D_{C\alpha C'}$ (●), and $^2D_{H\alpha C'}$ (●) RDCs, reported by Chou et al.(Chou *et al.* 2001) against values predicted by an SVD fit to the 1-Å X-ray structure (PDB entry 1EXR) (Wilson and Brunger 2000). (A-D) Individual SVD fits of normalized experimental RDCs in the four helices to the coordinates of the 1-Å X-ray structure (PDB entry 1EXR) (A-D) or to ideal helix (E-H). (A,E) helix 5 (E82-F92); (B,F) helix 6 (A102 to N111); (C,G) helix 7 (D118 to E127); and (D,H) helix 8 (Y138-M145).

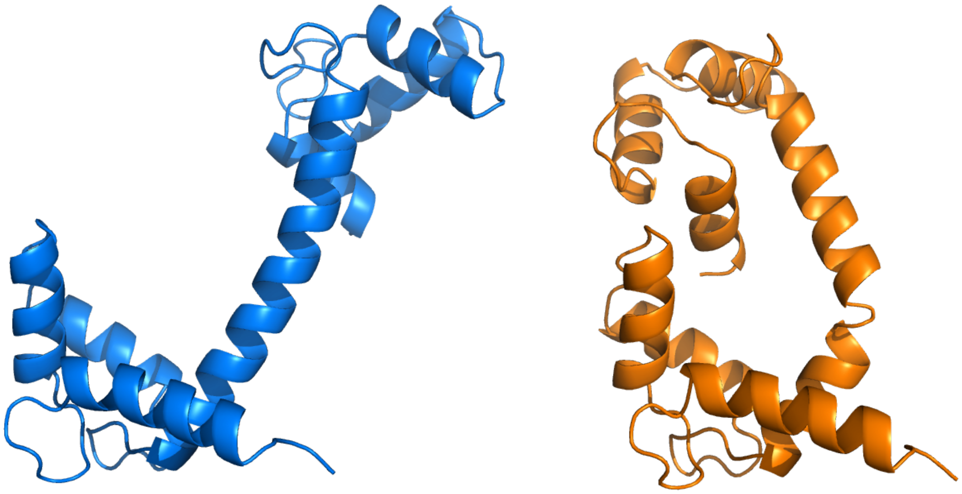

Figure S3. Ribbon diagram of the X-ray structure of Ca^{2+}-calmodulin (1EXR) before (left) and after (right) altering its C-terminal domain orientation such that the principal axes of the N- and C-terminal alignment tensors become parallel to one another.

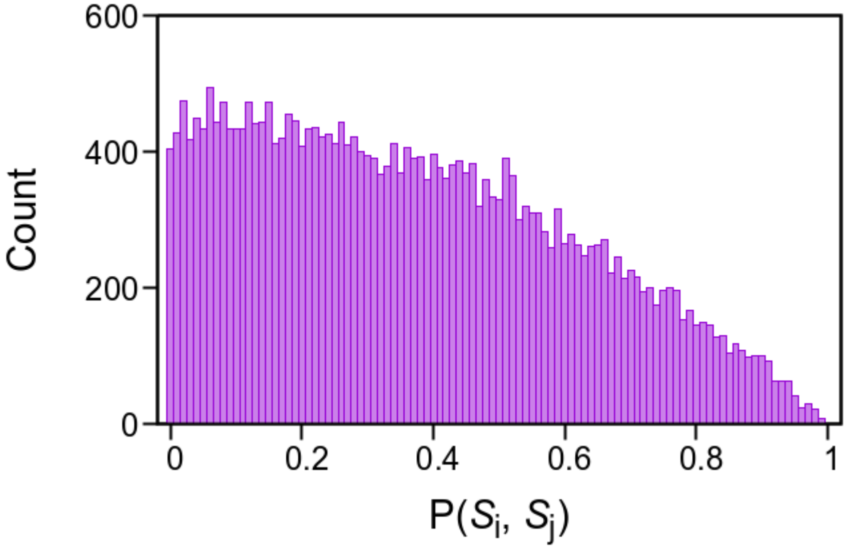

Figure S4. Histogram of normalized scalar products P(S_i, S_j) calculated for 30,000 randomly obtained alignment tensor pairs.

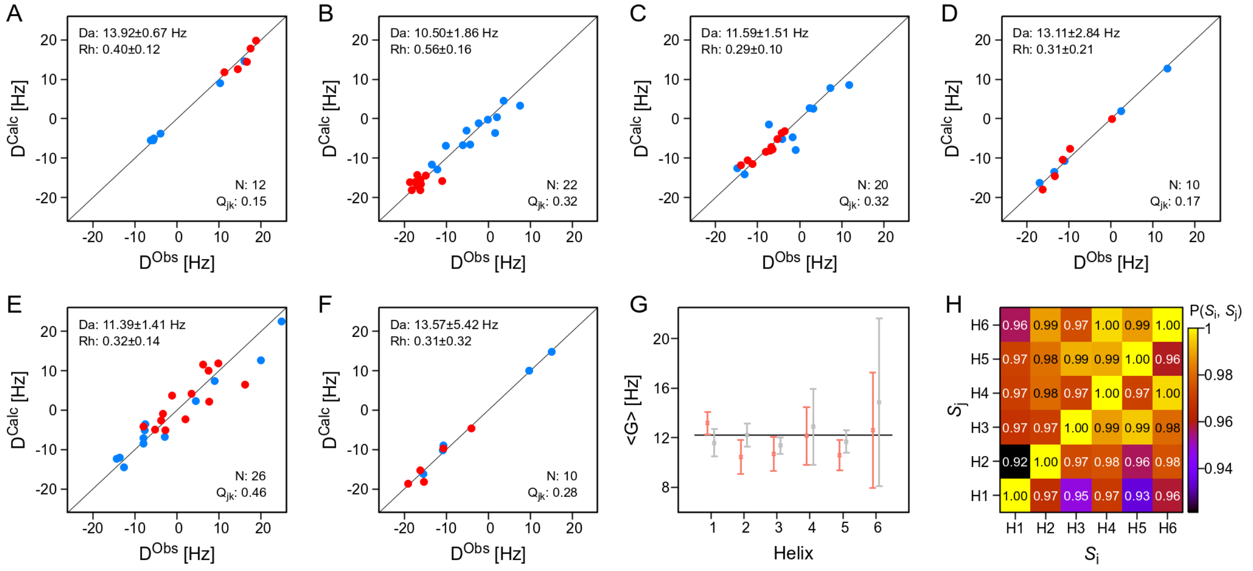

Figure S5. RDC analysis of α-helices in dimeric SARS-CoV-2 MPro. Fits of the normalized $^1D_{NH}$ (●), $^2D_{HC'}$ (●) RDCs to the X-ray coordinates of homodimeric MPro (PDB entry 7K3T) are shown for the following helices: (A) Y54-I59; (B) T201-I213; (C)L227-T237; (D) D245-L250; (E) V261-Q273; and (F) F294-Q299. The generalized sampling parameter for these RDCs, Ξ, is 0.33. (G) Generalized alignment strengths, *G*, obtained for the six helices when using 7K3T (grey) or idealized helices (orange) as reference structures; the reference G value of 12.20 obtained for all six helices (H1 to H6) is plotted as the horizontal line. (H) Normalized scalar products P(S_i, S_j) [i,j="H1" to "H6"] for the six alignment tensors *S* obtained when fitting the helical RDCs to the coordinates of the homodimeric X-ray structure (7K3T) (upper-left half), and to ideal helices that were best-fit superimposed on 7K3T (lower-right half).

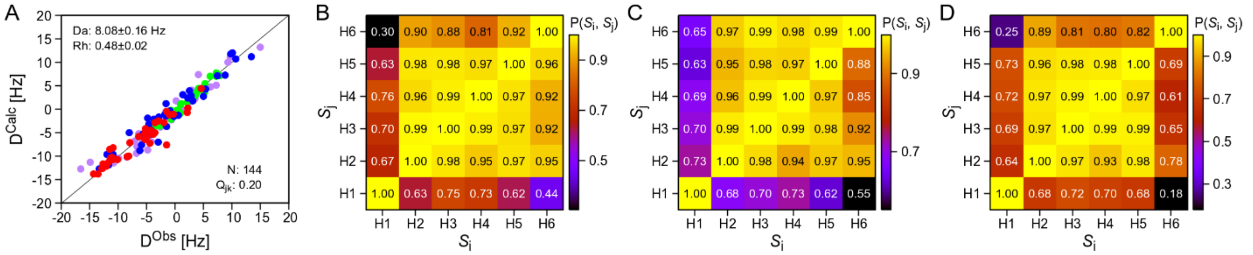

Figure S6. (A) Fits of the normalized $^1D_{NH}$ (●), $^1D_{C'N}$ (●), $^1D_{C\alpha C'}$ (●), and $^2D_{HNC'}$ (●) RDCs measured in the monomeric state to the X-ray coordinates of homodimeric MPro (PDB entry 7K3T) are shown for RDCs measured for the five helices in the C-terminal domain. (B-D) Normalized scalar products P(S_i, S_j) [i = "H1" to "H6"] for the six alignment tensors *S* obtained when fitting the RDCs to the actual X-ray coordinates of the helices in the monomeric X-ray structures (upper-left half) of (B) 2QCY, (C) 2PWX and (D) 3F9E, or to the best-fit superimposed individual idealized helices (lower-right half).

Table S1. Geometry parameters of the ideal helix used in this work.

ϕ	-62.8°
ψ	41.6°
ω	179.0°
H-N bond length[a]	0.99 Å
In-plane angle H_i-N_i-C^{α}_i	116.5°
Out-of-plane (C'_{i-1}-N_i-C^{α}_i) orientation	1.0°

[*] All other structural parameters follow the AMBER94 library (Cornell *et al.* 1995) used by MOLMOL (Koradi *et al.* 1996).

[a] This bond length provides optimal RDC fits for $^2D_{HNC'}$ (Robertson *et al.* 2021) and is not used for calculating the ^{1}H-^{15}N dipolar interaction constant that is librationally averaged.(Yao *et al.* 2008)

Table S2. Experimental D_{NH}, $D_{C'H}$, $D_{C'C}$, $D_{NC'}$, RDC values for the alpha helices of monomeric MPro$^{10-306,H41Q}$.

Residue Number	Residue Name	Atom	Residue Number	Residue Name	Atom	D (Hz)	Error[a] (Hz)
54	Tyr	N	54	Tyr	HN	-3.90	0.15
55	Glu	N	55	Glu	HN	3.77	0.20
56	Asp	N	56	Asp	HN	3.50	0.23
57	Leu	N	57	Leu	HN	-3.13	0.32
59	Ile	N	59	Ile	HN	5.11	0.16
201	Thr	N	201	Thr	HN	-3.81	0.37
202	Val	N	202	Val	HN	-5.27	0.22
203	Asn	N	203	Asn	HN	-11.10	0.32
204	Val	N	204	Val	HN	-4.25	0.58
227	Leu	N	227	Leu	HN	-11.37	0.13
229	Asp	N	229	Asp	HN	-14.38	0.20

Residue Number	Residue Name	Atom	Residue Number	Residue Name	Atom	D (Hz)	Error[a] (Hz)
230	Phe	N	230	Phe	HN	-11.72	0.18
231	Asn	N	231	Asn	HN	-10.94	0.18
232	Leu	N	232	Leu	HN	-11.32	0.22
233	Val	N	233	Val	HN	-13.58	0.38
234	Ala	N	234	Ala	HN	-11.29	0.37
235	Met	N	235	Met	HN	-4.95	0.25
236	Lys	N	236	Lys	HN	-11.47	0.33
237	Tyr	N	237	Tyr	HN	-12.86	0.40
245	Asp	N	245	Asp	HN	-1.49	0.13
246	His	N	246	His	HN	2.29	0.16
247	Val	N	247	Val	HN	-1.03	0.18
248	Asp	N	248	Asp	HN	-5.30	0.18
249	Ile	N	249	Ile	HN	-6.43	0.28
250	Leu	N	250	Leu	HN	4.65	0.40
261	Val	N	261	Val	HN	-3.53	0.11
262	Leu	N	262	Leu	HN	-10.10	0.18
263	Asp	N	263	Asp	HN	2.20	0.20
264	Met	N	264	Met	HN	-2.94	0.45
266	Ala	N	266	Ala	HN	-12.72	0.26
267	Ser	N	267	Ser	HN	-0.33	0.10
269	Lys	N	269	Lys	HN	-12.12	0.30
270	Glu	N	270	Glu	HN	-8.50	0.17
271	Leu	N	271	Leu	HN	-4.42	0.48
273	Gln	N	273	Gln	HN	-12.75	5.17
294	Phe	N	294	Phe	HN	-10.91	0.16
296	Val	N	296	Val	HN	-1.48	0.19
297	Val	N	297	Val	HN	-5.17	0.20
298	Arg	N	298	Arg	HN	-8.00	0.14
299	Gln	N	299	Gln	HN	-1.99	0.12
54	Tyr	N	53	Asn	C	-0.40	0.02
55	Glu	N	54	Tyr	C	-0.34	0.03
56	Asp	N	55	Glu	C	1.00	0.03
57	Leu	N	56	Asp	C	-0.58	0.04
201	Thr	N	200	Ile	C	1.30	0.05
202	Val	N	201	Thr	C	0.63	0.02
203	Asn	N	202	Val	C	-0.84	0.04
204	Val	N	203	Asn	C	1.30	0.08
227	Leu	N	226	Thr	C	0.95	0.02

Residue Number	Residue Name	Atom	Residue Number	Residue Name	Atom	D (Hz)	Error[a] (Hz)
228	Asn	N	227	Leu	C	0.89	0.01
229	Asp	N	228	Asn	C	0.01	0.02
230	Phe	N	229	Asp	C	0.90	0.02
231	Asn	N	230	Phe	C	0.07	0.02
232	Leu	N	231	Asn	C	1.93	0.03
233	Val	N	232	Leu	C	-0.66	0.04
234	Ala	N	233	Val	C	0.77	0.04
235	Met	N	234	Ala	C	0.26	0.03
236	Lys	N	235	Met	C	1.32	0.04
237	Tyr	N	236	Lys	C	0.19	0.04
246	His	N	245	Asp	C	-0.14	0.02
247	Val	N	246	His	C	1.03	0.02
248	Asp	N	247	Val	C	0.53	0.02
249	Ile	N	248	Asp	C	-1.27	0.04
250	Leu	N	249	Ile	C	1.95	0.06
261	Val	N	260	Ala	C	0.27	0.01
262	Leu	N	261	Val	C	0.87	0.02
263	Asp	N	262	Leu	C	-1.01	0.03
264	Met	N	263	Asp	C	1.82	0.06
266	Ala	N	265	Cys	C	-0.17	0.03
269	Lys	N	268	Leu	C	0.71	0.03
270	Glu	N	269	Lys	C	-0.66	0.02
271	Leu	N	270	Glu	C	1.55	0.07
273	Gln	N	272	Leu	C	0.76	0.02
294	Phe	N	293	Pro	C	1.04	0.02
296	Val	N	295	Asp	C	1.83	0.03
297	Val	N	296	Val	C	-0.17	0.02
299	Gln	N	298	Arg	C	0.21	0.02
53	Asn	C	53	Asn	CA	1.97	0.09
54	Tyr	C	54	Tyr	CA	0.61	0.10
55	Glu	C	55	Glu	CA	-1.65	0.11
56	Asp	C	56	Asp	CA	-0.33	0.14
200	Ile	C	200	Ile	CA	-1.26	0.22
201	Thr	C	201	Thr	CA	-1.19	0.13
202	Val	C	202	Val	CA	0.99	0.15
203	Asn	C	203	Asn	CA	1.08	0.20
226	Thr	C	226	Thr	CA	0.26	0.05
228	Asn	C	228	Asn	CA	-0.32	0.07

Residue Number	Residue Name	Atom	Residue Number	Residue Name	Atom	D (Hz)	Error[a] (Hz)
229	Asp	C	229	Asp	CA	0.62	0.08
230	Phe	C	230	Phe	CA	-1.25	0.07
231	Asn	C	231	Asn	CA	-0.97	0.08
232	Leu	C	232	Leu	CA	-0.85	0.12
233	Val	C	233	Val	CA	2.03	0.12
234	Ala	C	234	Ala	CA	-2.34	0.08
235	Met	C	235	Met	CA	-1.13	0.10
236	Lys	C	236	Lys	CA	0.51	0.12
244	Gln	C	244	Gln	CA	2.21	0.04
245	Asp	C	245	Asp	CA	-0.76	0.08
246	His	C	246	His	CA	-0.59	0.07
247	Val	C	247	Val	CA	-0.18	0.07
248	Asp	C	248	Asp	CA	1.76	0.14
249	Ile	C	249	Ile	CA	-0.31	0.16
260	Ala	C	260	Ala	CA	-2.81	0.05
261	Val	C	261	Val	CA	1.49	0.07
262	Leu	C	262	Leu	CA	0.57	0.09
263	Asp	C	263	Asp	CA	-0.86	0.18
264	Met	C	264	Met	CA	-2.19	0.15
265	Cys	C	265	Cys	CA	2.69	0.09
268	Leu	C	268	Leu	CA	0.28	0.08
269	Lys	C	269	Lys	CA	0.45	0.09
270	Glu	C	270	Glu	CA	-0.30	0.11
272	Leu	C	272	Leu	CA	2.14	0.08
293	Pro	C	293	Pro	CA	1.76	0.09
294	Phe	C	294	Phe	CA	0.13	0.08
295	Asp	C	295	Asp	CA	-0.57	0.07
298	Arg	C	298	Arg	CA	-0.61	0.04
53	Asn	C	54	Tyr	HN	-2.60	0.17
54	Tyr	C	55	Glu	HN	-0.25	0.22
55	Glu	C	56	Asp	HN	3.61	0.24
56	Asp	C	57	Leu	HN	-1.25	0.33
58	Leu	C	59	Ile	HN	2.74	0.14
200	Ile	C	201	Thr	HN	2.09	0.45
201	Thr	C	202	Val	HN	1.19	0.26
202	Val	C	203	Asn	HN	-4.99	0.38
203	Asn	C	204	Val	HN	0.77	0.36
226	Thr	C	227	Leu	HN	-0.51	0.09

Residue Number	Residue Name	Atom	Residue Number	Residue Name	Atom	D (Hz)	Error[a] (Hz)
227	Leu	C	228	Asn	HN	0.42	0.11
228	Asn	C	229	Asp	HN	-1.61	0.12
229	Asp	C	230	Phe	HN	-1.07	0.13
230	Phe	C	231	Asn	HN	-1.26	0.13
231	Asn	C	232	Leu	HN	2.42	0.14
232	Leu	C	233	Val	HN	-3.32	0.32
233	Val	C	234	Ala	HN	-2.13	0.27
234	Ala	C	235	Met	HN	0.05	0.16
235	Met	C	236	Lys	HN	1.72	0.22
236	Lys	C	237	Tyr	HN	-2.28	0.23
244	Gln	C	245	Asp	HN	-1.90	0.13
245	Asp	C	246	His	HN	-0.35	0.14
246	His	C	247	Val	HN	1.37	0.13
247	Val	C	248	Asp	HN	-0.46	0.14
248	Asp	C	249	Ile	HN	-5.54	0.33
249	Ile	C	250	Leu	HN	5.00	0.39
260	Ala	C	261	Val	HN	1.01	0.08
261	Val	C	262	Leu	HN	-0.85	0.14
262	Leu	C	263	Asp	HN	-2.37	0.18
263	Asp	C	264	Met	HN	3.25	0.27
264	Met	C	265	Cys	HN	-1.51	0.30
265	Cys	C	266	Ala	HN	-2.80	0.19
266	Ala	C	267	Ser	HN	0.98	0.20
268	Leu	C	269	Lys	HN	-1.41	0.17
269	Lys	C	270	Glu	HN	-3.60	0.20
270	Glu	C	271	Leu	HN	2.65	0.20
272	Leu	C	273	Gln	HN	-1.40	0.16
293	Pro	C	294	Phe	HN	-0.57	0.16
294	Phe	C	295	Asp	HN	-3.82	0.17
295	Asp	C	296	Val	HN	3.11	0.15
296	Val	C	297	Val	HN	-0.54	0.30
297	Val	C	298	Arg	HN	-2.22	0.13
298	Arg	C	299	Gln	HN	-0.18	0.09

[a] The error only accounts for the statistical uncertainty based on the signal to noise ratio in the reference and attenuated spectra (Fitzkee and Bax 2010) and does not include systematic errors that can result from baseline undulations or other factors that can impact peak intensities.

References

Chou JJ, Li S, Klee CB, Bax A (2001). Solution structure of Ca2+-calmodulin reveals flexible hand-like properties of its domains. Nat. Struct. Mol. Biol. 8: 990-997.

Cornell WD, Cieplak P, Bayly CI, Gould IR, Merz KM, Ferguson DM, Spellmeyer DC, Fox T, Caldwell JW, Kollman PA (1995). A 2nd generation force-field for the simulation of proteins, nucleic acid, and organic molecules. J. Am. Chem. Soc. 117: 5179-5197.

Fitzkee NC, Bax A (2010). Facile measurement of H-1-N-15 residual dipolar couplings in larger perdeuterated proteins. J. Biomol. NMR 48: 65-70.

Koradi R, Billeter M, Wuthrich K (1996). MOLMOL: a program for display and analysis of macromolecular structures. J. Mol. Graph. 14: 51-55.

Robertson AJ, Courtney JM, Shen Y, Ying JF, Bax A (2021). Concordance of X-ray and AlphaFold2 Models of SARS-CoV-2 Main Protease with Residual Dipolar Couplings Measured in Solution. J. Am. Chem. Soc. 143: 19306-19310.

Wilson MA, Brunger AT (2000). The 1.0 A Crystal Structure of Ca-bound Calmodulin: an Analysis of Disorder and Implications for Functionally Relevant Plasticity. J. Mol. Biol. 301: 1237-1256.

Yao L, Voegeli B, Ying JF, Bax A (2008). NMR determination of amide N-H equilibrium bond length from concerted dipolar coupling measurements. J. Am. Chem. Soc. 130: 16518-16520.
